# Business analytics approach to artificial intelligence

**DOI:** 10.3389/frai.2022.974180

**Published:** 2022-09-30

**Authors:** Melva Inés Gómez-Caicedo, Mercedes Gaitán-Angulo, Jorge Bacca-Acosta, Carlos Yesid Briñez Torres, Jenny Cubillos Díaz

**Affiliations:** ^1^Economic Sciences, The Liberators University, Bogotá, Colombia; ^2^Business School, Konrad Lorenz University Foundation, Bogotá, Colombia; ^3^Faculty of Mathematics and Engineering, Pilot University of Colombia, Bogotá, Colombia; ^4^Economics and Management, University Corporation of Meta, Villavicencio, Colombia

**Keywords:** artificial intelligence, business analytics, economics and business, economics, grow

## Abstract

Artificial Intelligence has become an essential element for strengthening the business fabric. The advances obtained in recent years as a result of the incorporation of technology for the improvement of productive activities and the positioning of companies in the markets are remarkable. Hence, the purpose of this paper is to analyze the origin, evolution and development of business analytics (BA) and its relationship with Artificial Intelligence (AI); from the conceptualization, evolution and identification of the main characteristics and research areas of AI and BA, as well as research conducted and published in journals indexed in Scopus between 2002 and 2022. The aim is to define the incidence of BA in business activities and analyze scientific activity and advances of BA to define new research horizons in this field. For this purpose, a bibliometric and documentary analysis is applied, allowing to highlight the findings that provide recognition and comparison of the results. This will facilitate the understanding of the current dynamics, its importance for organizations, and its impact in the face of the new challenges generated by the requirements of world trade.

## Introduction

The term business analytics is relatively new. Initially, it was presented as a concept related to economics and the way of managing resources. Business analytics can be defined as the process of collecting data, processing this data and gain insights from the data (Gupta, 2021). Business analytics is also defined as the science of discovering insights from data to support timely decision-making (Delen and Ram, 2018).

In the 1950s when time and motion studies began to be used in production processes, and the 1960s when computers were used for automatic data processing (Delen and Ram, 2018), analytics was used for the use of mathematical models to provide a solution to the problem. Analytics is used for the use of mathematical models to provide solutions to problems identified in organizations (Sharma, 2016).

In this way, Business analytics uses statistical and mathematical models to respond to problems or needs in organizations. Simon (2017) states that analytics is the process of using raw data to obtain clues and improve the understanding of a topic or phenomenon.

Frequently in the literature, it is also clarified that Business analytics cannot be considered as report generation, because the latter is limited to the visualization of the behavior of one or several variables, while the former gives answers to questions of interest to an organization around its business (Simon, 2017).

Camm et al. (2020) consider that business analytics is “the scientific process of transforming data into clues for making better decisions” (p. 5).

For Muñoz-Hernandez et al. (2016) business analytics allows the efficient use of all the information available to organizations, to obtain a competitive advantage. Other authors such as Evans and Lindner (2012) consider that it is fundamental for decision making.

Thus, Zumstein et al. (2022) in their research highlight the increase in the level of maturity, benefits, challenges and development of companies and economies from the use of artificial intelligence.

Likewise, authors such as Shi et al. (2022), indicate that a large amount of data and the limits of digital products drive organizations to use business analytics (BA) to increase customer engagement. Within their main findings, they indicate that BA culture does not directly improve performance on its own but must be integrated with existing organizational strengths. In addition, the development of the literature on this subject shows that BA techniques moderate the relationship between customers and innovation since it allows companies to be better informed when data is available online.

This has led several companies to choose to have in their structure an area dedicated exclusively to business analytics and the relationship between research and data analysis processes, which contribute to the improvement of productive conditions (Acito and Khatri, 2014).

Silva et al. (2021) indicate that the term “Industry 4.0” has emerged to characterize various adoptions of Information and Communication Technologies (ICT) in production processes. Hence, business analytics (BA) is considered as a technological advancement that facilitates decision making (Namvar et al., 2021). Thus, business analytics facilitates data analysis and relates it to the use of emerging technologies, which enable the transformation of decision-making dynamics in organizations (Seufert and Schiefer, 2005; Ward et al., 2014).

Resource management starts from the premise that technology and data analytics are at the service of operational efficiency, which allows organizations to understand their information and use this analysis to identify problems and decisions.

During the last decades, the dynamics of markets and companies have generated conditions that strengthen productive activity, facilitating the development of processes that tend to strengthen productive activity. Hence, several elements have been identified that, when used efficiently, promote growth and competitiveness, such as resource management based on the premise that technology and data analytics are at the service of operational efficiency, which allows organizations to understand their own information and use this analysis to identify problems and decisions.

In that regard, business analytics uses statistical analysis, predictive modeling, data mining and other techniques to employ information and develop a competitive advantage in its favor (Evans and Lindner, 2012; Medina, 2012).

The link between business analytics and artificial intelligence can be seen from different perspectives. One the one hand, artificial intelligence is considered to be one of the three pillars of business analytics together with visualization and statistical modeling (Raghupathi and Raghupathi, 2021). In particular, the machine learning subset of artificial intelligence is the most common component of this pillar of business analytics. Machine learning provides the techniques and methods to gain insights from business data. On the other hand, artificial intelligence is also considered to be the evolution of traditional analytics and the era of artificial intelligence is called analytics 4.0 in the context of business analytics (Davenport, 2018). Moreover, other authors suggest that business analytics is often supported by artificial intelligence to transform data into information (Schmitt, 2022).

Hence, the objective of this research is to analyze the evolution and development of artificial intelligence and its relationship with Business Analytics, based on its conceptualization, evolution, identification of its main characteristics, research areas and the recognition of publications indexed in Scopus between 2002 and 2022. In this sense, in the first part of this document a systematic and historical review of business analytics is made, in the second part the main publications associated with this concept from 2001 to 2018 are presented, the most cited authors, the countries that are most interested in the subject, and finally, how research networks have been created from its relationship with Artificial Intelligence.

### Historical analysis of business analytics

To understand the origin of business analytics it is important to understand how statistics and mathematics have been used throughout history as a support for the development of competitive intelligence in organizations.

During the industrial revolution, statistics began to be used in standardization and manufacturing processes as a control tool. Additionally, the focus of organizations begins to be the minimization of waste and therefore the optimization of production costs, becoming a trend and consolidating as the quality movement (Quality Movement) (Sharma, 2016). From this movement emerged several years later practices such as Six Sigma and Toyota's just-in-time manufacturing methodology.

Years later, when the United States participated in World War I, quality and standardization become fundamental aspects of production processes because ammunition had to be compatible with weapons from different manufacturers and countries. At this time, organizations begin to invest in Total Quality Management training and statistical measurement processes (van Kemenade and Hardjono, 2018), allowing the emergence of various techniques such as control charts, histograms, Pareto and scatter diagrams (Sadeghi Moghadam et al., 2021).

Toward the decade of the 1920s, the quality control method is known as Statistical Process Control also emerged as a mechanism to control production processes seeking their best standardization and with the minimum possible waste (Zan et al., 2019).

Likewise, historical data began to be used for climate prediction. In 1950 the first numerical weather prediction was developed, performed on the ENIAC computer by a group of meteorologists and mathematicians. In 1956 engineer Bill Fair and mathematician Earl Isaac found Fair Isaac Corporation (FICO) as a company to use data intelligently for the development of competitive intelligence (Sharma, 2016). Two years later they launch their credit risk and scoring system for investments in the United States.

In 1958, in the IBM Journal of Research and Development, an article written by Luhn is published where one of the initial references to the term Business Intelligence is made. This article proposes the construction of an intelligent system that uses data processing mechanisms to perform auto-summarization and auto-coding of documents to provide different information profiles according to the organization's lines of action (Luhn, 1958).

During the 1960s most companies began to use centralized systems for inventory control and in the 1970s guidelines were developed to facilitate materials planning. It should be noted that, during this period, data collection was done annually, and the available data came from manual processes through interviews and questionnaires from which mathematical models could be built to solve optimization problems with constraints. In this way, those problems that could not be solved with linear and non-linear models were addressed through simulations (Delen and Ram, 2018).

In the 1970s, Rule-Based Enterprise Systems also emerged with the promise that the knowledge of an expert in a specific domain could be represented as a set of rules that could be processed by a machine and could be used to solve queries as an expert would (Simões et al., 2020).

Subsequently, in the 1980s, Enterprise Resource Planning (ERP) or Enterprise Resource Planning (ERP) systems emerge and become the first data collection and storage systems for organizations to provide support in areas such as planning, sales, manufacturing, distribution and costs (Sharma, 2016).

Thus, the emergence of relational database systems enabled the capture, storage and organization of data to avoid duplication. At this time, the amount of data being stored was larger and one of the major challenges was to maintain data integrity and consistency.

This is how the concept of enterprise data warehouses or Enterprise Data Warehouse (EDW) emerged as unified data storage systems for organizations. These systems were also upgraded so that they could respond to various changes in data effectively to display information in real time l which gave rise to real-time data warehouse systems (Delen and Ram, 2018).

In this regard, EDWs facilitated the collection of data from different sources which was subsequently used to extract knowledge and information of interest to organizations and this gave rise to the term Business Intelligence (BI) in the first decade of the 2000s, initially focusing on the analysis of data collected by organizations to know the progress of the organization.

The 1990s also saw the emergence of executive information systems, i.e., decision support systems, which displayed information using graphs and charts to facilitate decision making (Delen and Ram, 2018).

Sharma (2016) states that between the years 1990 and 2000 organizations began to see the need to use the data obtained to be able to generate predictive analytics, through descriptive, inferential, differential and associative statistical techniques. Hence, the amount of data produced by users or consumers through devices and interaction with social networks and other digital media led to the emergence of a new term: big data, which refers to techniques and procedures to analyze large amounts of unstructured data and the emergence of methods such as “Deep learning.”

It is important to note that the term business analytics has been associated with other terms such as Business Intelligence and Supply Chain Management that have been in the research spotlight for some years. However, the studies derived from each of the terms differ in the analysis and results obtained. For example, Supply Chain Management was one of the central topics in business research during the decade from 2000 to 2005. However, this term was transformed into Business Intelligence, because conducting research focused solely on the Supply Chain left aside other types of information.

Business Intelligence was born as a response to the lack of information from organizations for the analysis of existing dynamics. In addition, the decrease in the costs of data storage services has increased the volume of data that organizations keep and this has allowed the growth of Business Intelligence as a fundamental area for organizations. It has been estimated that the storage volume to be reached by 2020 will be 40 zettabytes (1,021 bytes or 1 sextillion bytes) (Sharma, 2016).

Today, some researchers are still being conducted that ensure that the predictive value of Business Intelligence interferes with the natural dynamics of information coming from organizations. Therefore, business analytics generates new information from company data without creating new data, but rather by analyzing existing data.

Based on this premise, business analytics studies indicate that there is a close relationship between the use of data analysis and the performance of an organization in terms of revenue, competitiveness, profitability and shareholder return. This means that entities with better performance are those in which the use of data analysis is an extra component compared to their competitors and this gives them a greater probability of strengthening their competitiveness (Davenport and Harris, 2007; Evans and Lindner, 2012).

The results suggest a statistically significant relationship between organizations' competencies have analytics on performance and the effect of business process-oriented information systems. The results provide a better understanding of the areas where the impact of business analytics may be the strongest (Trkman et al., 2010).

### Related works

Previous works that have attempted to synthesize research in the area of business intelligence can be found in the literature. For example, Gimenez et al. (2015) conducted a systematic review of 22 articles on the applications of business analytics in the supply chain. The authors report the main challenges and trends in the area. Similarly, Mishra et al. (2018) conducted a bibliometric analysis on the topics of Big Data and Supply Chain Management between 2006 and 2016, analyzing 280 publications in the 20 most important journals in the area.

However, these two references only allow us to appreciate the research is done in business analytics (BA) in its relationship with Supply Chain. Recently, Yin and Fernandez (2020) conducted a systematic literature review (40 articles in the area of BA) to present a common definition, its applications, research methods and its relationship with Business Intelligence (BI). The authors include a bibliometric review focused on the evolution of publications between 2000 and 2018, as well as the journals where the topic is most published and the most relevant authors. However, the bibliometric review is focused only on articles that have received a certain number of citations, which means that the results do not fully reflect the overall BI landscape.

Sahoo (2021) conducted a bibliometric review of 89 articles focusing on the terms Big Data and BA as they relate to the topic of “manufacturing.” In this article, the authors identify several areas of future work and research challenges. However, although the reported results are valuable to the scientific community, the bibliometric review is focused solely on the area of manufacturing.

Similarly, Silva et al. (2021) conducted a systematic literature review of 169 articles to identify the relationship between business analytics and Industry 4.0. From this literature review, the authors conclude that there are still many open questions surrounding its application.

The research was conducted by Dahish (2021), who conducted a literature review of 57 articles on Business Intelligence and social networks; and authors such as Purnomo et al. (2021) who reviewed Scopus databases on the same subject in the period between 1975 and 2020 stand out. The research was focused on identifying relevant topics in the area.

Ting-Peng and Yu-Hsi (2018) conducted a bibliometric literature review with a broader focus on articles indexed in Web of Science on the topics of Big Data and Business Intelligence during the years 1990 and 2017. One of the main findings emerged much earlier than the concept of Big Data, publications have grown faster and are associated with algorithmic and computational topics, while the area of Business Intelligence is more associated with management, data analytics and predictive analytics topics.

Zumstein and Kotewski (2020) indicate in their research that digital commerce is growing in most countries, it is a medium used by new and established online retailers to promote their different products, digital and customer services are considered essential to increase business success. Within the results of this study we found, that digital analytics is important to study and monitor digital business. By analyzing different success factors, this technique contributes to online stores having customer, service and data orientation generating high conversion rates and revenues. Finally, successful omnichannel marketers use various digital marketing channels, such as search engine optimization and advertising, marketing.

Chiang et al. (2018) highlight in their research the importance of the correct accumulation of data for analysis, decision making and strategic planning in organizations, based on the design and application of different analytical techniques since it is concluded that data analysis without generating value offers no contribution to organizations, regardless of whether the data is big or small.

## Methodology

Bibliometrics emerged in the field of library and information science, allowing statistical and quantitative analysis of scholarly outputs, including descriptive statistics, networks on keywords, texts, citations, authors, institutions and their connections. This methodology allows establishing the frequency, connectedness, centrality, author and text group, publication trends, knowledge base, citation pattern, author network, reader usage, impact and importance of a topic or article (Huang et al., 2017).

For there to be conceptual clarity, scientific research must be associated with an exhaustive review of the area of knowledge to be worked on to be clear about the possible areas for the development of the research and its difficulty. For this, there is the bibliometric review, a quantitative analysis technique that uses mathematical and statistical methods to know the main characteristics of the topic under consideration (Ejdys, 2016; Sarkar and Searcy, 2016).

This article will carry out a historical review of artificial intelligence that will provide the basis for developing a descriptive bibliometric analysis that allows us to synthesize and understand the evolution of business analytics in certain fields of knowledge. For this analysis, publications published between 2002 and 2022 will be reviewed, in the Scopus bibliographic database, in the indexed academic literature that addresses specific topics directly related to the subject.

The first stage shows the route used to carry out the bibliometric study was:

(TITLE-ABS-KEY (“business analytics”) OR TITLE-ABS-KEY (“business analytics”) OR TITLE-ABS-KEY (“analyse des affaires”) OR TITLE-ABS-KEY (“análise de negócios”) OR TITLE-ABS-KEY (“analisi aziendale”)) AND (EXCLUDE (PREFNAMEAUID, “Undefined#Undefined”)) AND (LIMIT-TO (DOCTYPE, “cp”) OR LIMIT-TO (DOCTYPE, “ar”) OR LIMIT-TO (DOCTYPE, “ch”) OR LIMIT-TO (DOCTYPE, “re”) OR LIMIT-TO (DOCTYPE, “bk”)) AND (EXCLUDE (PUBYEAR, 2023))).

The search filters consisted of the terms “business analytics,” which could be located in the title, abstract and keywords; the search was conducted in English, Spanish, French, Italian, Portuguese and French; it was limited to conference papers, articles, book chapters, reviews and books and was limited to the period from 2002 to 2022. The search yielded 1,605 documents.

The purpose of the second stage was to analyze the information using the *Bibliometrix* package of R. This was done to visually organize the information downloaded from Scopus and to obtain schemas that would feed the research carried out.

Finally, in stage 3, the analysis of the descriptive results was carried out, where the information obtained was condensed, relating the evolution of artificial intelligence together with business analytics and the results obtained.

**Figure 1:**
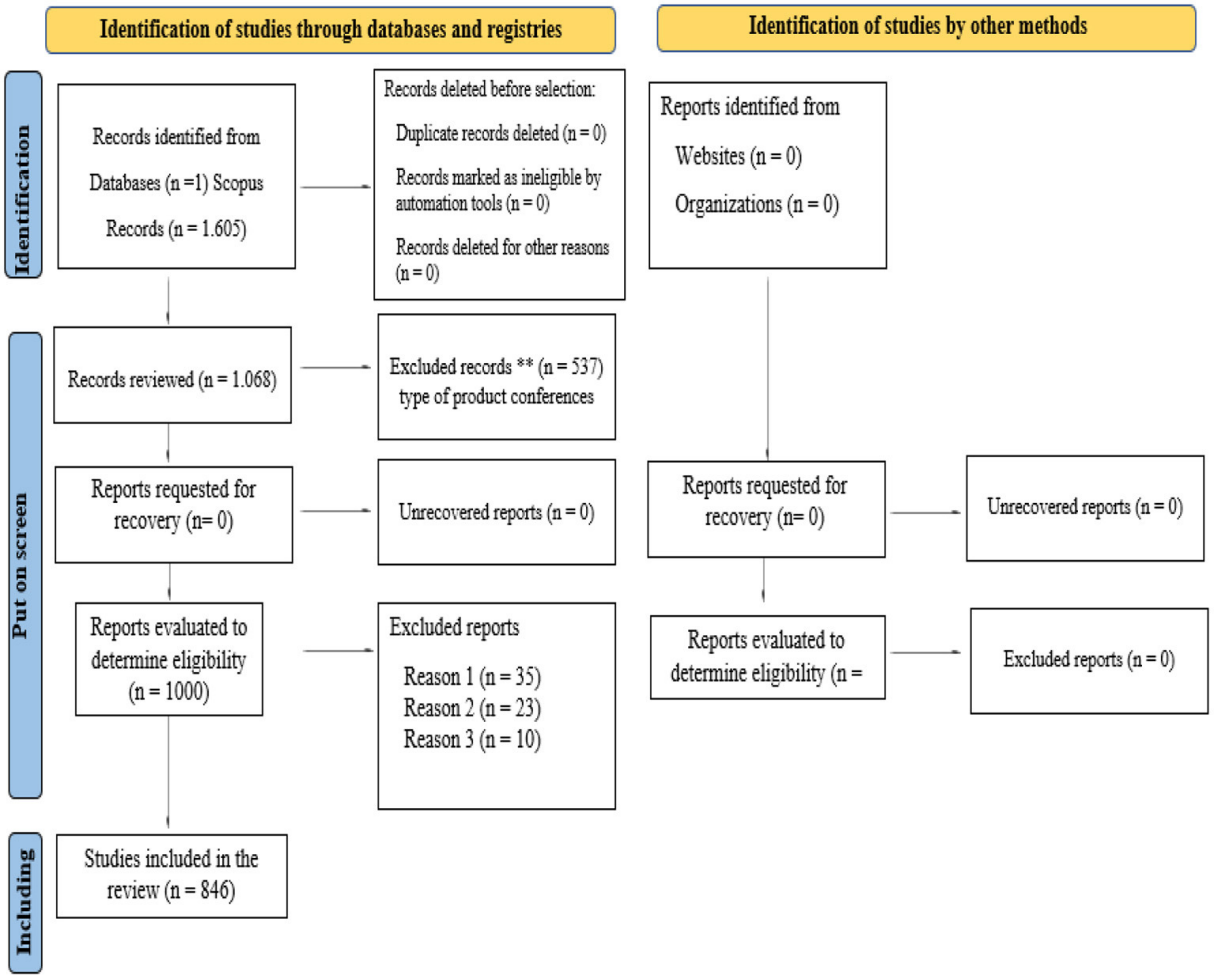
PRISMA. Source: Own elaboration using Prisma Statement 2020. http://www.prisma-statement.org/. Source: Page et al., 2021.

Figure 1 shows how the articles included in this bibliometric review were selected.

### Descriptive results

From the results of the search, it can be noted that business analytics has been a topic of interest to researchers since 2002. It is possible to make this inference since the information searches do not yield results from before that year.

**Figure 2:**
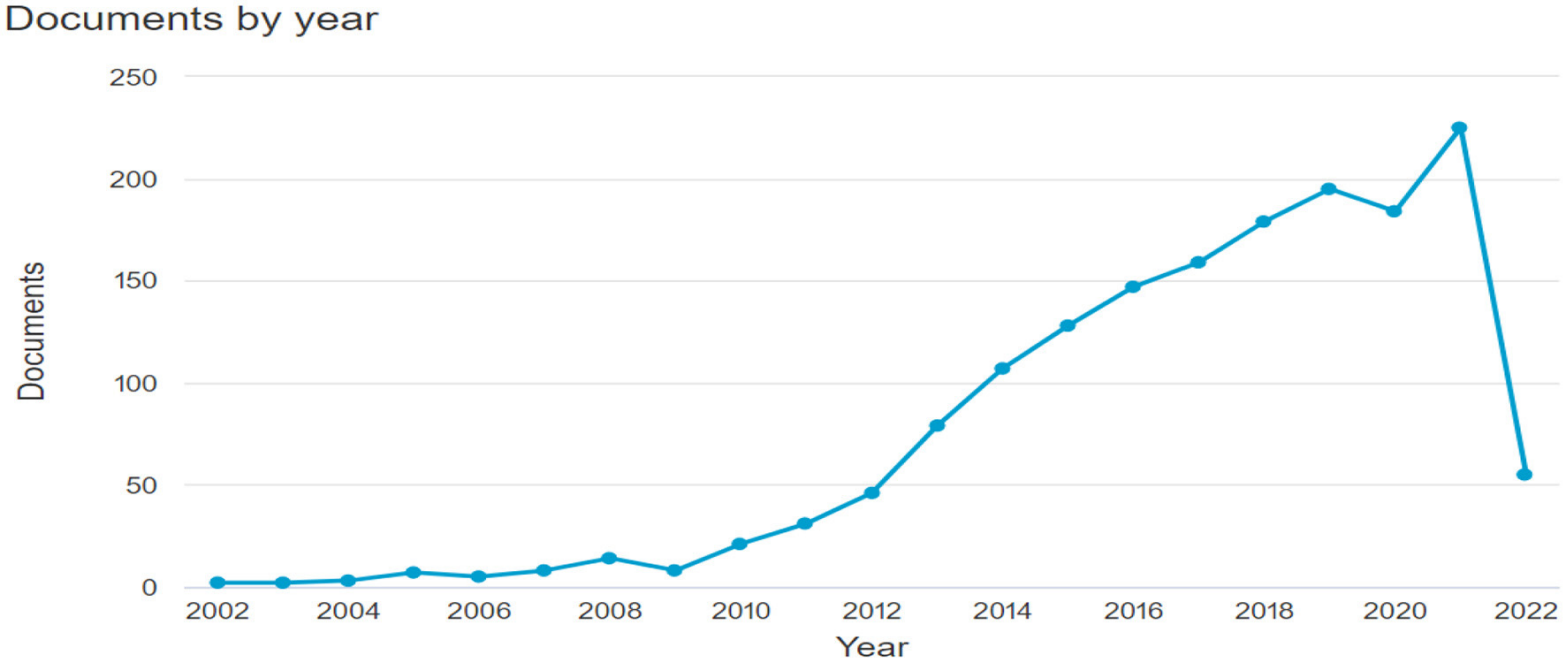
Annual scientific production. Source: Own elaboration using Bibliometrix.

In addition, it was possible to identify that the number of research projects is increasing and has reached 210 publications in 2021 alone (see Figure 2; Table 1).

**Table 1:** Production per year.

**Year**	**Documents**
2022	115
2021	228
2020	189
2019	197
2018	181
2017	159
2016	148
2015	129
2014	107
2013	83
2012	46
2011	31
2010	21
2009	8
2008	14
2007	8
2006	5
2005	7
2004	3
2003	2
2002	2

Source: Own elaboration using Bibliometrix.

**Figure 3:**
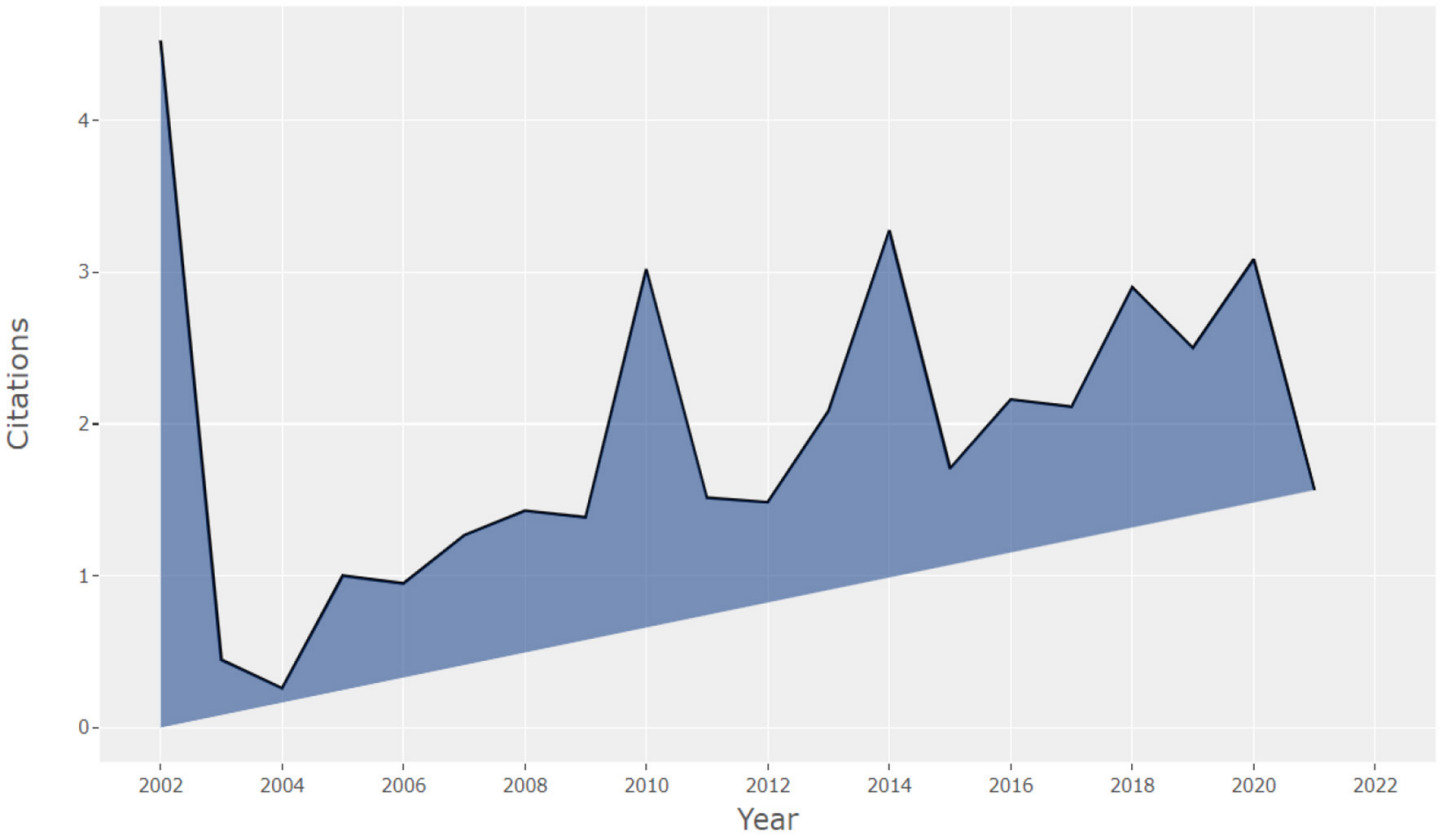
Average number of article citations per year. Source: Own elaboration using Bibliometrix.

Moreover, not only the annual output indicates the evolution of the term, other factors such as the average number of citations of articles per year indicate the value of the academic output and its usefulness in another research (see Figure 3).

Among the findings of the search, it is possible to point out that the articles produced in 2002 are those that have been most cited by other researchers. Likewise, it can be observed that in 2004 there was a non-significant number of citations, while from 2005 onwards they increase, with two peaks of higher citations between 2010 and 2014. This tendency may be due to the changes and topics published in those years (see Figure 6).

**Figure 4:**
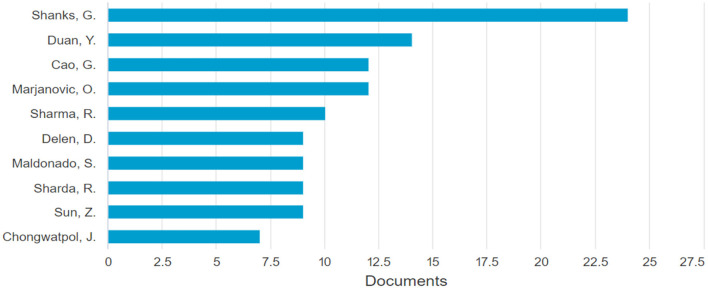
Most relevant authors. Source: Own elaboration using Bibliometrix.

It was also possible to identify the authors who have published the most papers on the subject: Shanks with more than 20 papers associated with business analytics, followed by Duan with ~10 papers and Cao, Marjanovic, and Sharma with eight papers each (see Figure 4; Table 2).

**Table 2:** Production by authors.

**Author**	**Documents**
Shanks, G.	24
Duan, Y.	14
Cao, G.	12
Marjanovic, O.	12
Sharma, R.	10
Sun, Z.	10
Delen, D.	9
Maldonado, S.	9
Sharda, R.,	9
Chongwatpol, J.	7
Oztekin, A.	7

Source: Own elaboration using Bibliometrix.

**Figure 5:**
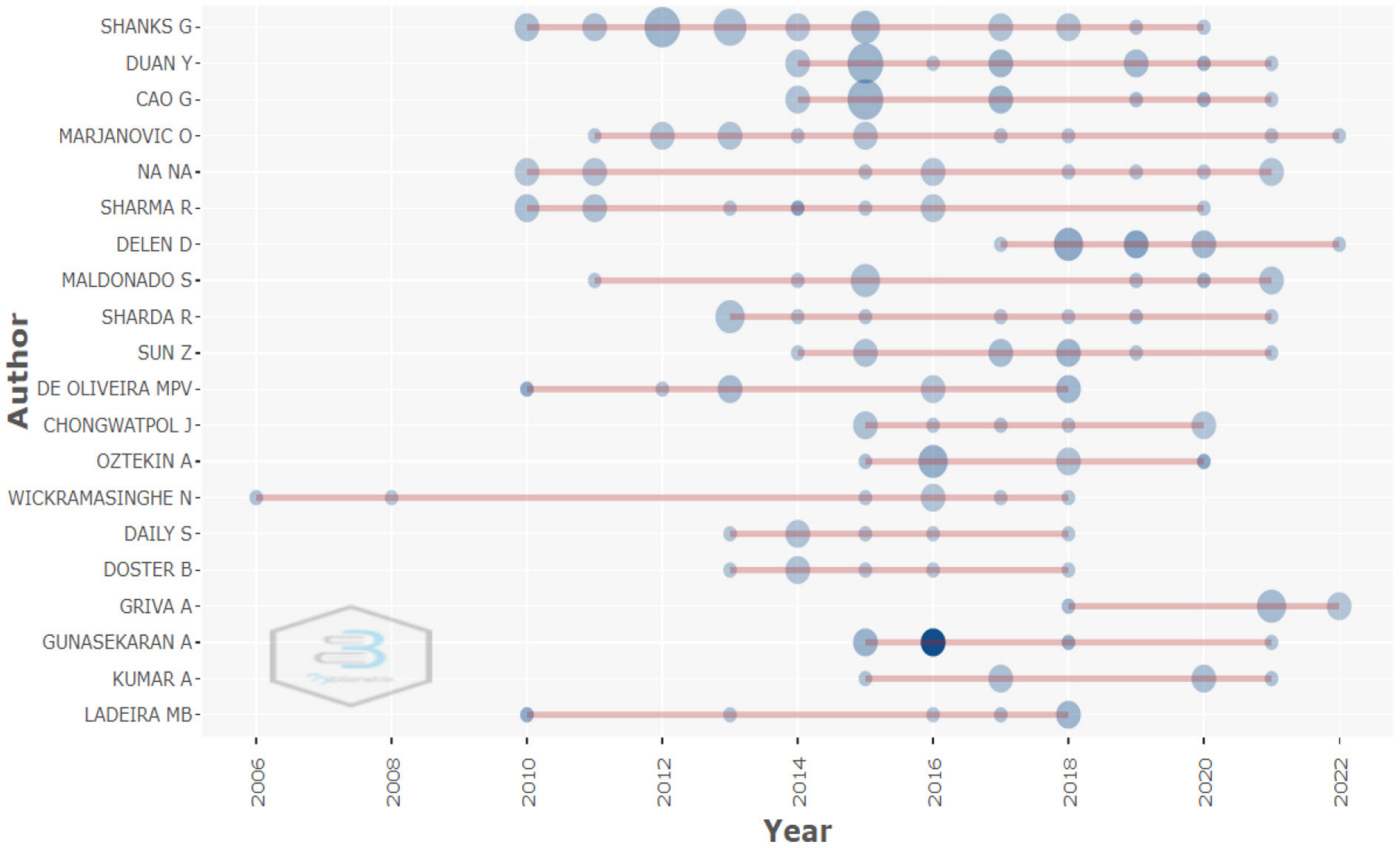
Production of the main authors by year. Source: Own elaboration using Bibliometrix.

In addition, the results allow us to analyze the number of publications made by the authors according to the year of publication (see Figure 5), which shows that there are authors such as Shanks G., De Oliveira MPV, Na Na, who maintain their production levels between 2010 and 2022, with some insignificant variations per year. The graph shows the number of publications, with the larger the circle indicating the author and the year, the higher the author's level of production. For example, Bekmamedova had a high volume of publications in 2012, but in 2013 it decreased and in the following years there were no publications. Also, authors such as Sharda R., Daily S. Doster B. Ryan J. and Lewis C. started research on the subject in 2013 and have maintained the publication trend until 2022.

**Figure 6:**
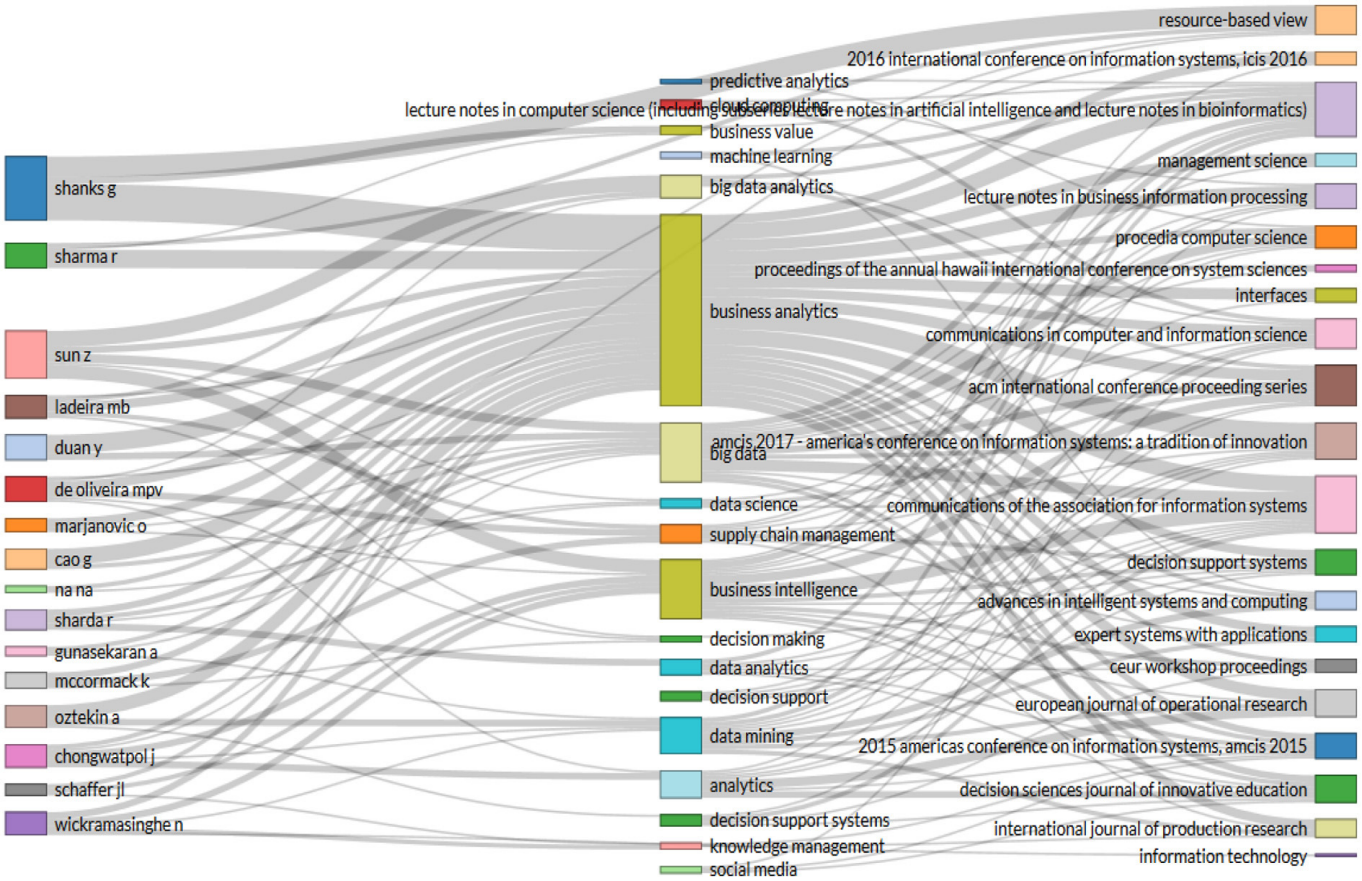
Graph of three fields: Authors, subjects, and journal names. Source: Own elaboration using Bibliometrix.

One of the reasons why authors such as Shanks have remained significant authors in business analytics is due to the consistent level of publications per year (see Figure 5). It is also important to note that the relationship that exists between authors, the topics to be covered and the journals that publish these topics are the central dynamic that ensures the success of business analytics as a major Research Topic (see Figure 6).

For example, Shanks, the author with the most publications (see Figure 4), has researched topics related to business analytics and predictive analytics, publishing in journals such as Resource-based View. On the other hand, Duan and Cao, the second and third authors respectively, have worked on business analytics and Big Data and have published in journals such as Communications of the association for information systems and Communications in computer and information science.

**Figure 7:**
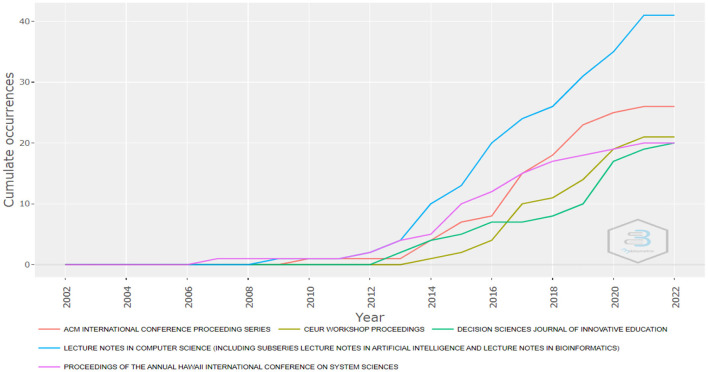
Growth of publication sources. Source: Own elaboration using Bibliometrix.

Regarding the dynamics of the publication sources, the results showed similar indicators to the period in which the topic had the highest number of publications. For example, in 2010, publications began to increase (Figure 2) and the main publication sources also began to increase the level of publications on business analytics. The source with the highest growth is AMCIS 2017—Americas conference on information systems: A tradition of innovation. The second fastest growing source is ACM International Conference Proceeding Series (see Figure 7). However, most of the sources show a similar development and between 2010 and 2012 the growth was much more significant.

**Figure 8:**
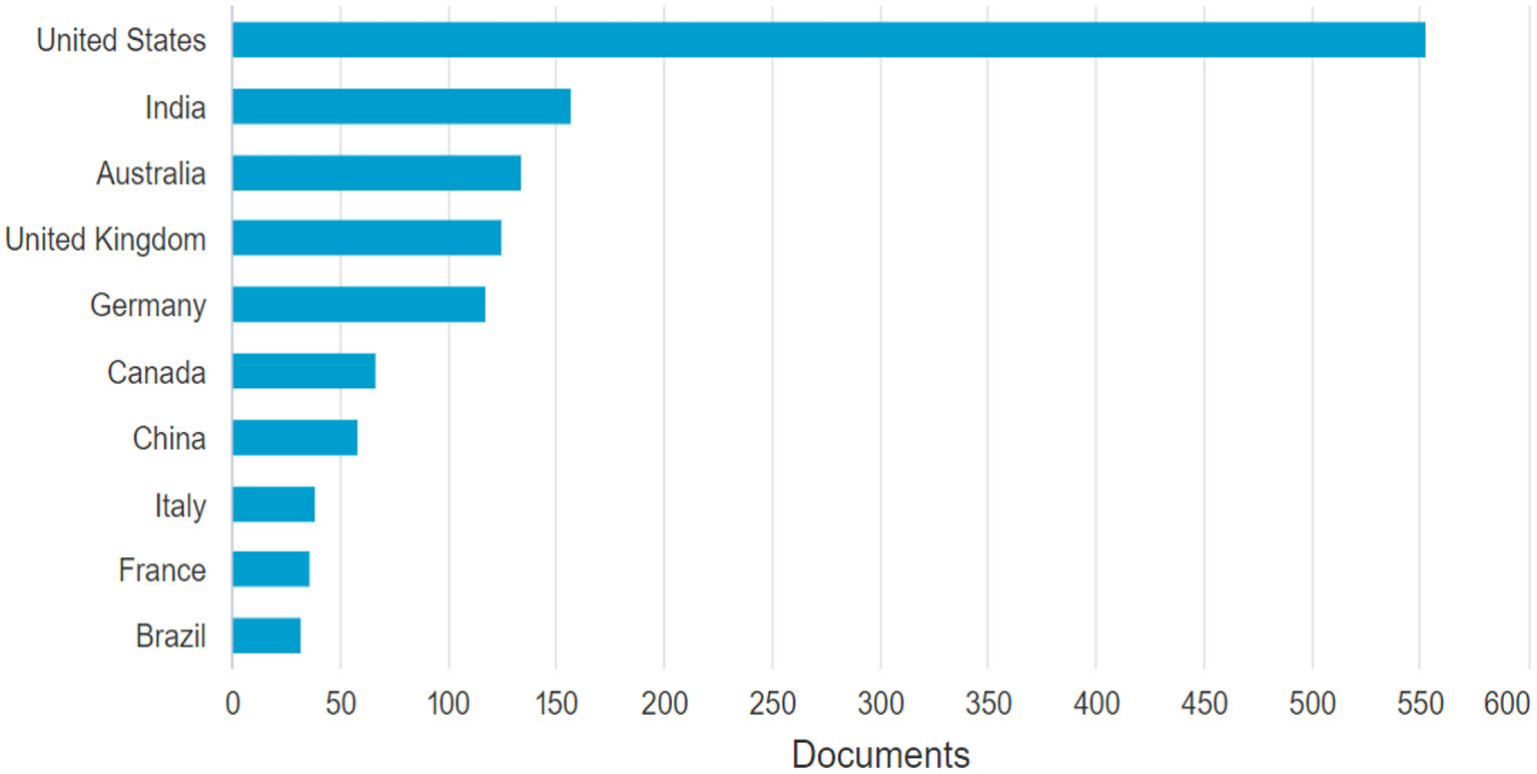
Countries of publication. Source: Own elaboration using Bibliometrix.

The relationship between sources and countries of the publication provides clues about the dynamics of researchers. For example, the United States is the country with the highest number of publications with ~2,400 publications. This contrasts significantly with the second country, Australia, which has ~400 publications (see Figure 8; Table 3). The difference is significant and exposes the importance of the United States for business analytics.

**Table 3:** Most cited countries.

**Country**	**Total citations**	**Average article citations**
USA	4,594	18.83
United Kingdom	1,080	21.18
Slovenia	459	91.80
Singapore	168	21.00
Spain	113	10.27
Switzerland	98	19.60
Sweden	82	13.67
Thailand	77	9.62
Poland	63	4.85
Portugal	37	3.70
Turkey	20	4.00
Romania	20	6.67
Saudi Arabia	17	3.40
South Africa	15	3.75
Qatar	13	13.00
Ukraine	12	3.00

Source: Own elaboration using Bibliometrix.

It is also significant to note that Brazil is the only Latin American country to appear in the top 20 ranking for business analytics publications.

**Figure 9:**
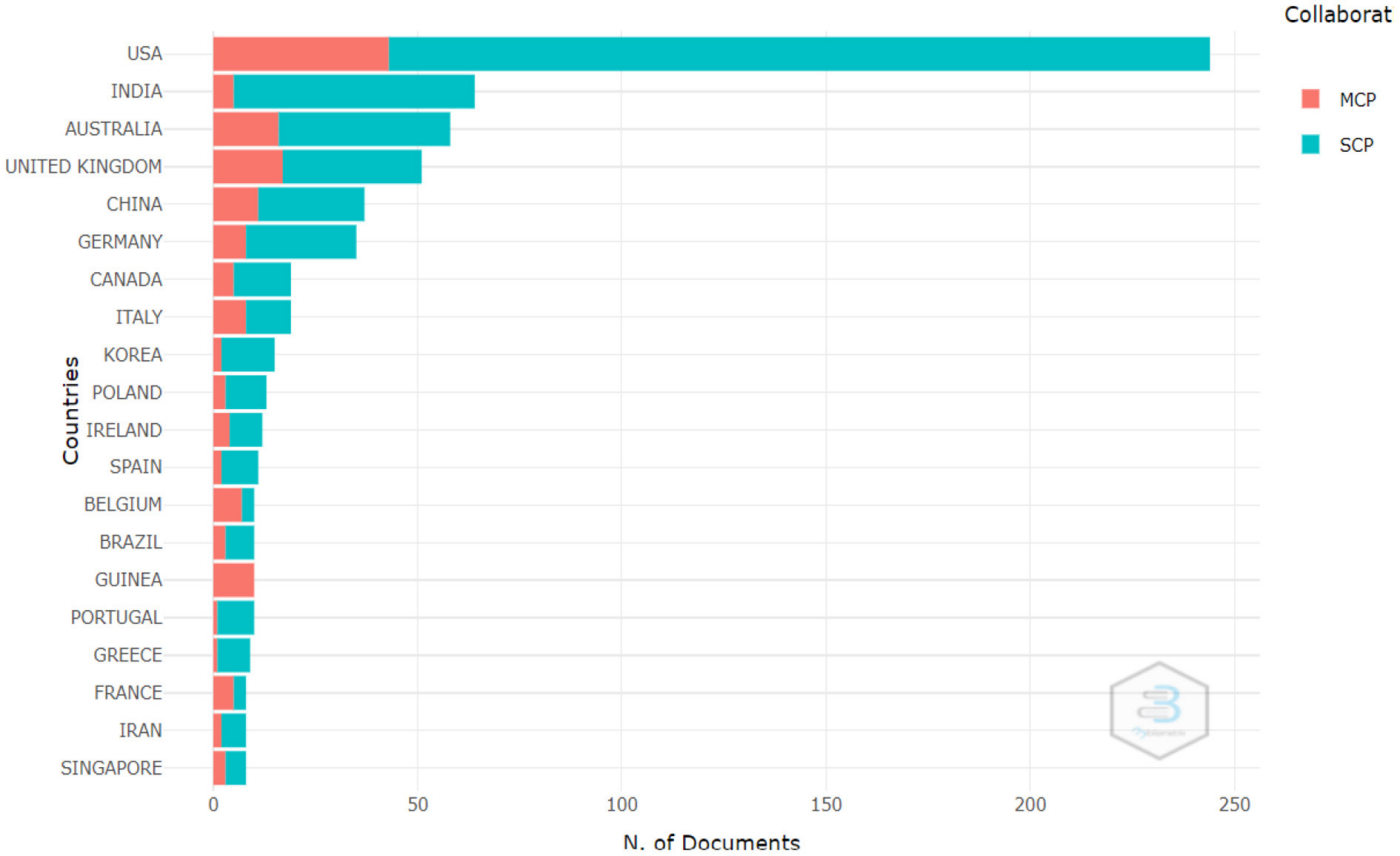
Correspondence between author and country. Source: Own elaboration using Bibliometrix.

However, most of the publications produced by the countries are international collaborations. Figure 9 shows how the dynamics of co-authored publications are generated. In green are the publications of Multiple Country Publications and in orange are the publications of the Single Country Publications type. It is possible to point out that collaborative publications have a greater impact and significantly position the country in the publication rankings.

**Figure 10:**
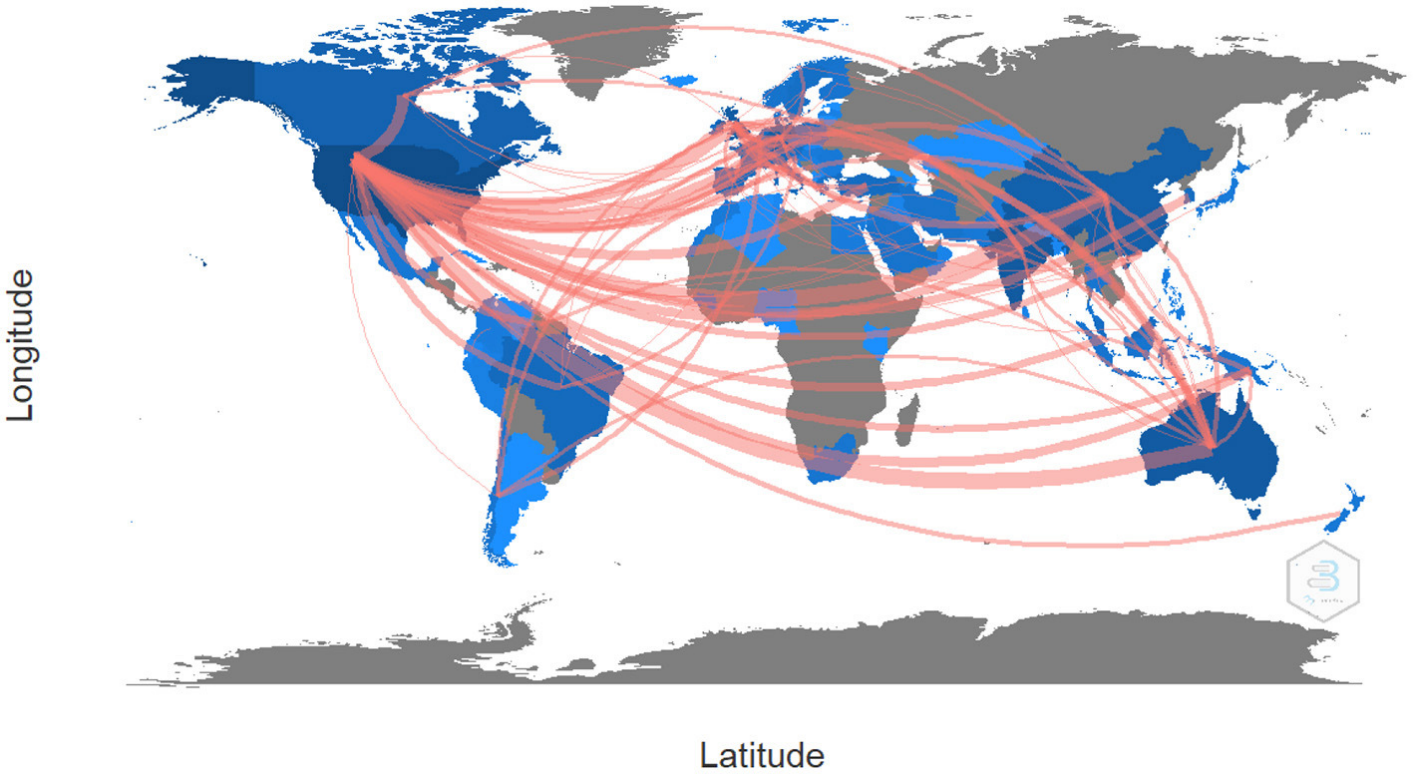
Map of collaboration between countries. Source: Own elaboration using Bibliometrix.

It is also important to note that partnerships between countries are vital to accounting for the research development of business analytics. The results show that the United States is the country with the highest number of collaborations. Within its relationships are countries such as France, Spain, Italy, Germany, China, Brazil, India, Portugal, Norway, Sweden and Finland, which are the countries in dark blue (see Figure 10). The strongest connection between countries is between the United States and Australia with a combined frequency of 21 citations. Countries such as New Zealand, South Africa, Egypt, Saudi Arabia, Nigeria, Colombia, Chile and Canada are countries with a medium number of publications on business analytics and mostly have partnerships with the United States and Australia. Finally, the countries in gray are those with no publications in this area.

**Figure 11:**
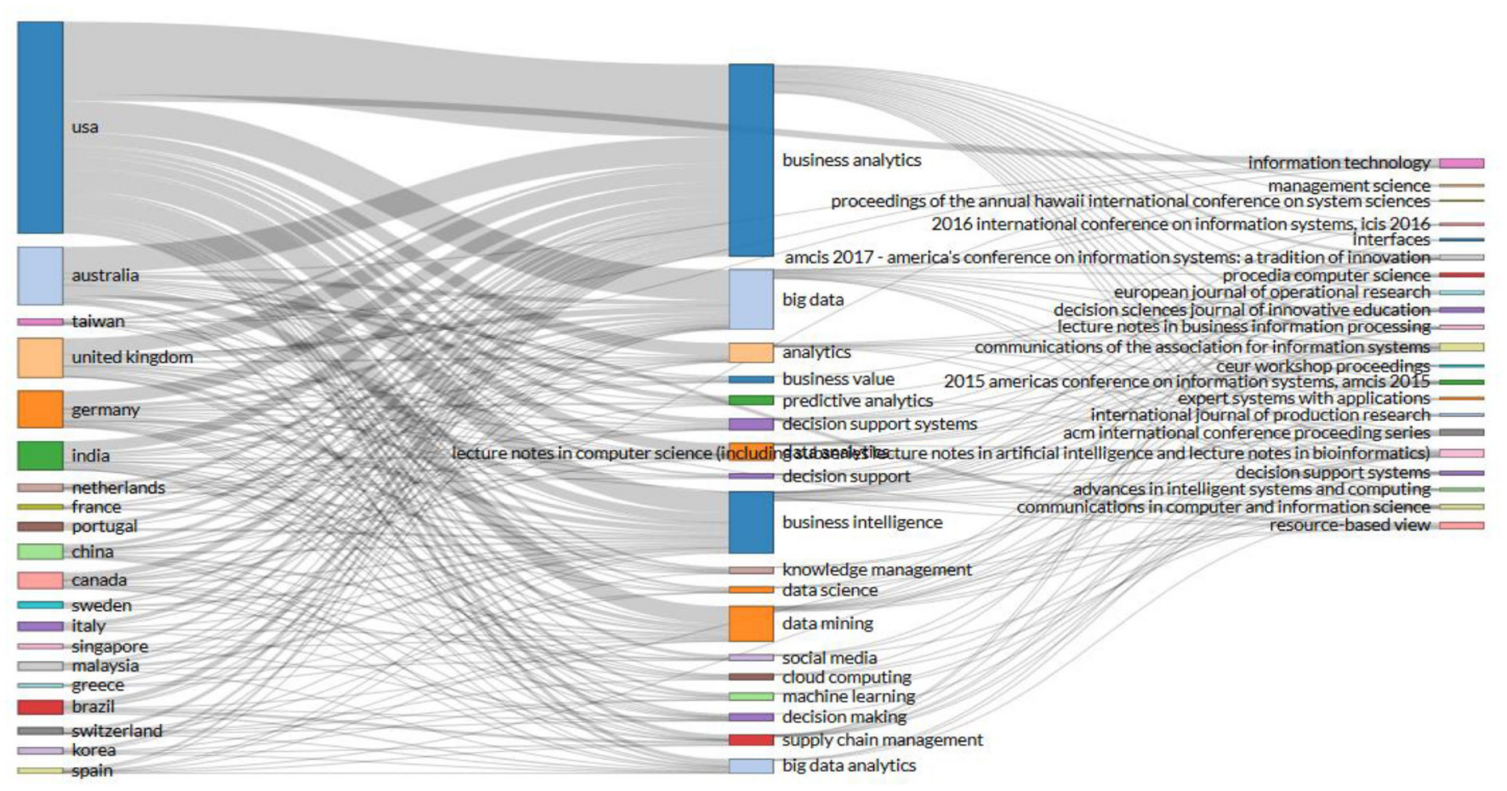
Three-Fields Plot Keyword Chart. Source: Own elaboration using Bibliometrix.

Within these country associations, there are common themes that help to relate and understand what the topics of interest are according to the country, the keywords and the journal of publication. For example, the United States has worked on business analytics related to Big Data, business value, predictive analytics, business intelligence, data mining, social media, predictive analytics, among others, with the journal Information Technology having the largest number of publications (see Figure 11).

On the other hand, Australia remains the second country in the ranking of publications and works on topics such as big data, social media, knowledge management, decision making, support systems, among others similar to those researched in the United States, hence the strong relationship that exists between the countries concerning publishing partnerships.

**Figure 12:**
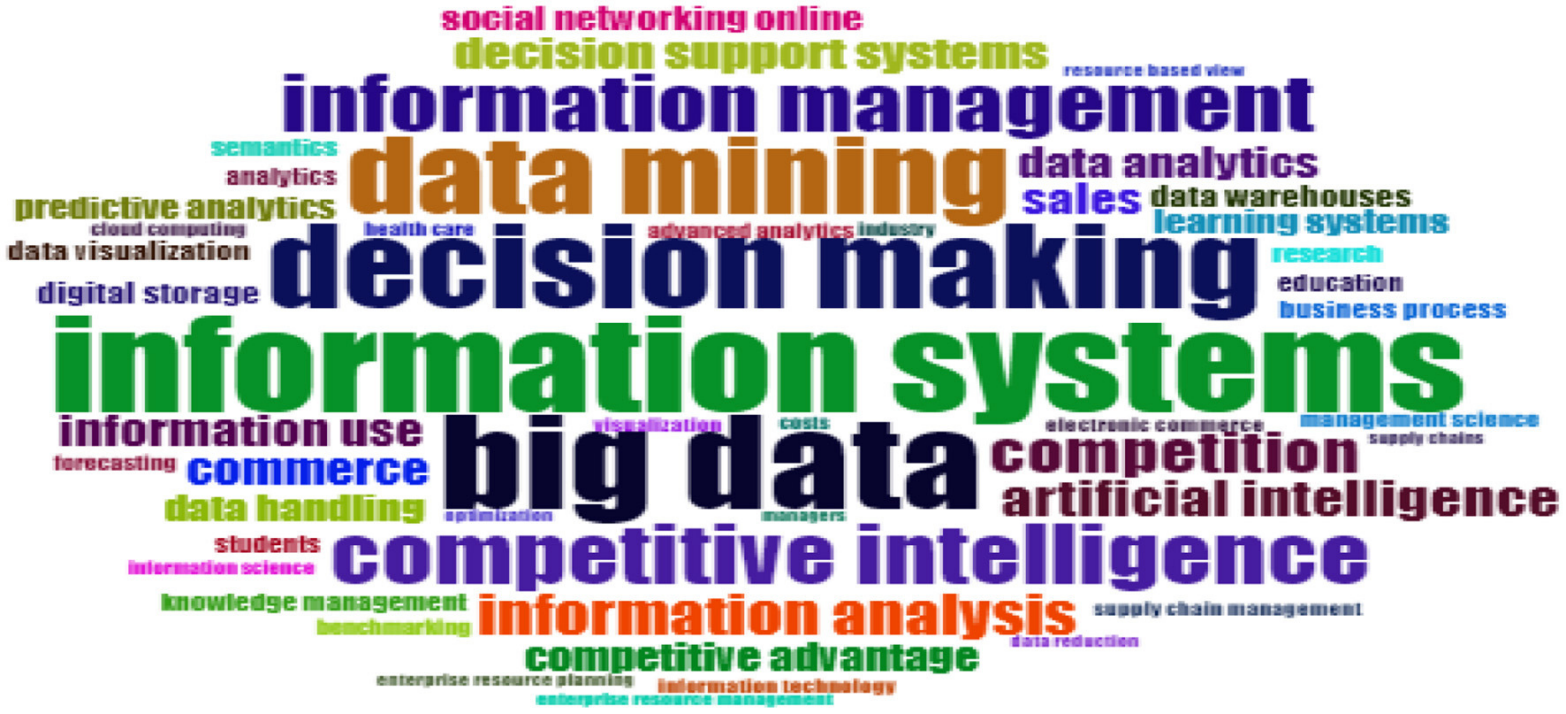
Keywords. Source: Own elaboration using Bibliometrix.

Thus, according to the topics that are most related to business analytics, it can be observed that data mining, decision marketing, information systems, Big Data and competitive intelligence are the topics that are most related and with which business analytics has been most researched (see Figure 12).

**Figure 13:**
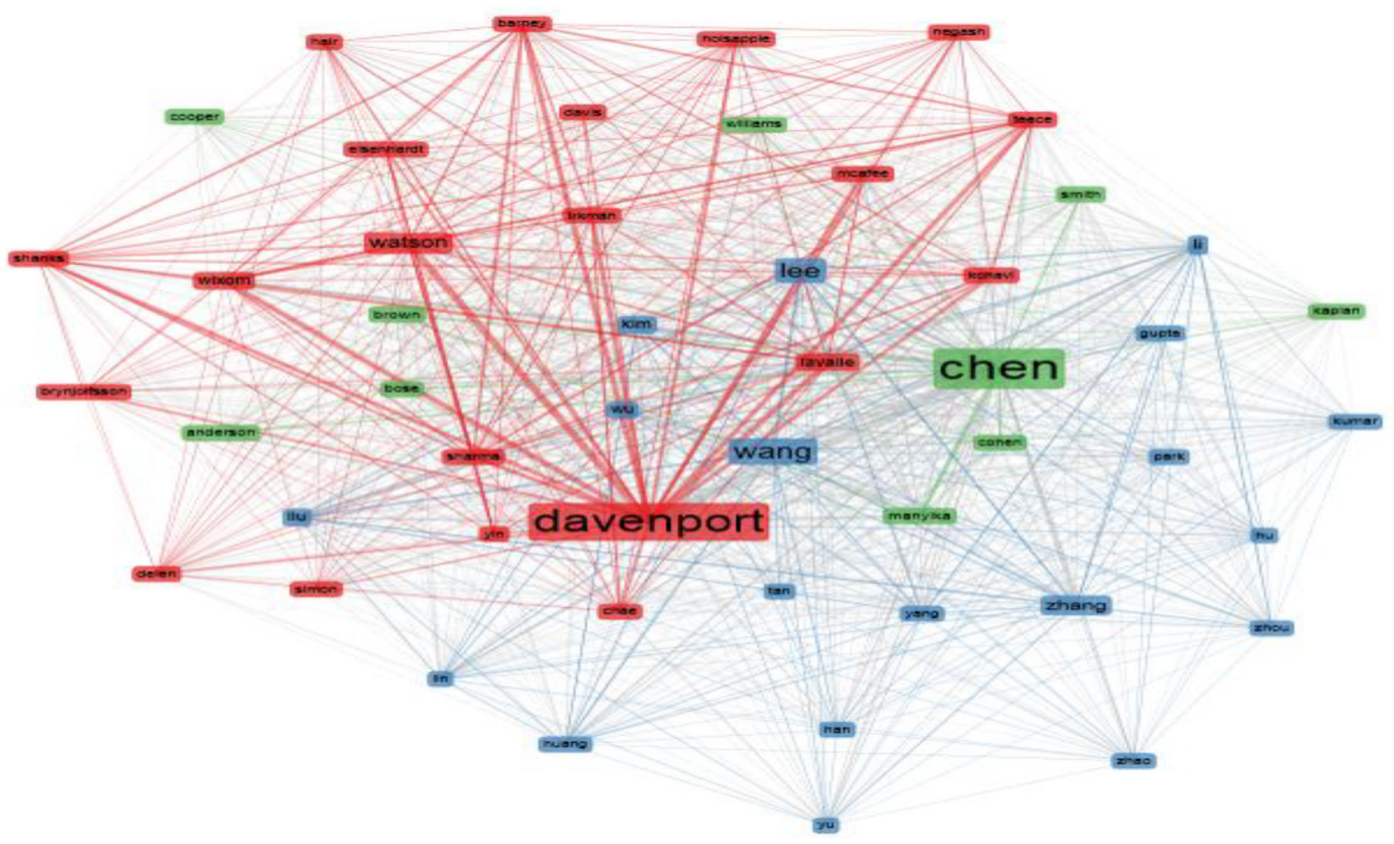
Co-citation networks. Source: Own elaboration using Bibliometrix.

The search results indicate that there are co-citation networks between authors that determine the alliances that exist for business analytics research. For example, in Figure 13 it can be seen that there are three co-citation networks (indicated in blue, red and green) which are distinguished from each other by the connections established.

One of the largest networks is identified with red color, it is composed of authors such as Davenport, Watson, Shanks, Lavalle, Simon, Yin, among others, which are shown related to the citation lines between them. The second significant network is identified with blue and is composed of authors such as Wang, Zhan, Yang, Wu, Han, Lee, Kim among others, it is an extensive network, however, the composition of the figure in terms of size and position it can be established that it has less impact than the red network. Finally, there is the green network, which is smaller, but is immersed between the red and blue networks, and is composed by authors such as Chen, Cohen, Anderson, Manykil, Bose, among others.

## Discussion

Business Intelligence is a tool that allows organizations to take advantage of all the information of competitive advantages in the market and decision making, as by combining its analysis with emerging technologies, it allows information to be obtained and projects possible situations that may arise in the development of the productive activity. The results of this bibliometric analysis show that there is an increasing interest in the field because the number of publications is growing every year. This result confirms the findings of previous studies that reported an exponential growth in the number of publications in this field (Yin and Fernandez, 2020). However, business analytics is still an emerging field (Raghupathi and Raghupathi, 2021) and further research is needed to uncover its affordances and benefits for a timely deciation to improve the conditions in which resources are used. Hence, its relationship with business analytics facilitates the generation-making support in companies.

With the results obtained throughout this research, it was possible to establish the influence of AI and BA on productive development. It should be noted that the trend of growth in the number of research and publications to be generated will increase and contribute significantly to the improvement of the business and competitive fabric of economies.

The development of AI and BA research in the United States stands out as reported in previous studies in the field of business analytics (Yin and Fernandez, 2020), followed by Australia, Germany, India, the United Kingdom, and Canada. However, unlike previous studies in the field, in this paper we identified that India is another country that is publishing research in the field of business analytics and is currently in the second position in the most productive countries in this field.

The topics that commonly appear connected with the term business analytics are: data mining, decision marketing, information systems, big data and competitive intelligence. This result shows that other field such as big data and artificial intelligence are relevant for the implementation of business analytics approaches in companies around the world. In that regard, the support of different fields is important to overcome some challenges that business analytics face today such as the need to collect data from multiple sources and process them effectively and in real-time so that the results can be used for making decisions and void the lag between data collection and data analysis (Raghupathi and Raghupathi, 2021).

Another topic that appeared connected with business analytics was descriptive analytics, which is one of the three types of analytics often reported in the literature. The other two types of analytics (predictive and prescriptive analytics) did not appeared frequently in this bibliometric analysis. A possible interpretation of this result might be that research on descriptive analytics has captured the attention of researchers in the first era of business analytics. However, further research is needed to investigate the use of predictive and prescriptive analytics for decision-making processes at companies. Moreover, recent research suggest a new type of business analytics: discovery analytics. This later type of analytics might be considered the next step in business analytics and is focused on supporting the discovery of new markets, products and strategies (Raghupathi and Raghupathi, 2021).

The results also show that there are three co-citation networks on this topic, one of the largest networks is composed of authors such as Davenport, Watson, Shanks, Lavalle, Simon, Yin, the second of authors such as Wang, Zhan, Yang, Wu, Han, Lee, Kim among others, and the third of authors such as Chen, Cohen, Anderson, Manykil, Bose, among others.

## Conclusions

This paper presents an overview of the research landscape in business analytics. We found that business analytics is an emerging field that has attracted the attention of many researchers around the world and the number of publications is increasing year by year. To further develop this field and increase the impact of this field in the industry, there is a need of a synergy between scholars and companies to identify the best practices in business analytics that are effective for companies.

Future research directions in the field of business analytics include the investigation of the impact of predictive, prescriptive and discovery analytics through case studies to uncover the affordances and benefits of these types of analytics for the timely decision-making of companies around the world. Moreover, it is important to continue working in this line of knowledge as we have seen the benefits of this topic, we can continue to deepen in topics such as digital analytics, due to the importance for companies to study and improve their campaigns, user experience, search engine marketing and the achievement of digital business objectives.

## Data availability statement

The raw data supporting the conclusions of this article will be made available by the authors, without undue reservation.

## Author contributions

All authors listed have made a substantial, direct, and intellectual contribution to the work and approved it for publication.

## Conflict of interest

The authors declare that the research was conducted in the absence of any commercial or financial relationships that could be construed as a potential conflict of interest.

## Publisher's note

All claims expressed in this article are solely those of the authors and do not necessarily represent those of their affiliated organizations, or those of the publisher, the editors and the reviewers. Any product that may be evaluated in this article, or claim that may be made by its manufacturer, is not guaranteed or endorsed by the publisher.
